# Evolutionary Analysis of Placental Orthologues Reveals Two Ancient DNA Virus Integrations

**DOI:** 10.1128/jvi.00933-22

**Published:** 2022-10-27

**Authors:** Jose Gabriel Nino Barreat, Aris Katzourakis

**Affiliations:** a Department of Biology, University of Oxford, Oxford, United Kingdom; Cornell University

**Keywords:** comparative genomics, endogenous viral elements, EVEs, *Homo sapiens*, paleovirology

## Abstract

The genomes of eukaryotes preserve a vast diversity of ancient viruses in the form of endogenous viral elements (EVEs). Study of this genomic fossil record provides insights into the diversity, origin, and evolution of viruses across geological timescales. In particular, *Mavericks* have emerged as one of the oldest groups of endogenous viruses infecting vertebrates (≥419 million years [My]). They have been found in the genomes of fish, amphibians, birds, and nonavian reptiles but had been overlooked in mammals. Thus, their evolutionary history and the causes of their demise in mammals remain puzzling questions. Here, we conducted a detailed evolutionary study of two *Maverick* integrations found on human chromosomes 7 and 8. We performed a comparative analysis of the integrations and determined their orthology across placental mammals (Eutheria) via the syntenic arrangement of neighboring genes. The integrations were absent at the orthologous sites in the genomes of marsupials and monotremes. These observations allowed us to reconstruct a time-calibrated phylogeny and infer the age of their most recent common ancestor at 127 to 262 My. In addition, we estimate the age of the individual integrations at ~102 My, which represents the oldest nonretroviral EVEs found in the human genome. Our findings suggest that active *Mavericks* still existed in the ancestors of modern mammals ~172 My ago (Jurassic Period) and potentially to the end of the Early Cretaceous. We hypothesize that *Mavericks* could have gone extinct in mammals from the evolution of an antiviral defense system or from reduced opportunities for transmission in terrestrial hosts.

**IMPORTANCE** The genomes of vertebrates preserve a large diversity of endogenous viral elements (remnants of ancient viruses that accumulate in host genomes over evolutionary time). Although retroviruses account for the vast majority of these elements, diverse DNA viruses have also been found and novel lineages are being described. Here, we analyzed two elements found in the human genome belonging to an ancient group of DNA viruses called *Mavericks*. We studied their evolutionary history, finding that the elements are shared between humans and many different species of placental mammals. These observations suggest that the elements inserted at least ~102 million years ago (Mya) in the most recent common ancestor of placentals. We further estimated the age of the viral ancestor at around 127 to 262 My. Our results provide evidence for some of the oldest viral integrations in the human genome and insights into the ancient interactions of viruses with the ancestors of modern-day mammals.

## INTRODUCTION

Viruses are highly diverse and abundant infectious agents that persist in hosts by exploiting two main strategies: (i) horizontal transmission between cells in the host population and (ii) vertical transmission between dividing cells, either by integrating into the host genome or by persisting as an extrachromosomal nucleic acid (episome) (1–3). Vertical transmission involves a state of stable association with the host cell in which viral particles (virions) are not produced. In the integrated state, endogenous viruses resemble transposons, which also colonize the host genome and can be transferred vertically and horizontally (4). However, endogenous viruses are distinguished from transposons by the capacity to reactivate and form virions. Therefore, endogenous viruses retain a conserved gene module involved in the formation of virions, the capsid morphogenesis module, which is common to all viruses and absent in transposable elements (3, 5).

The genomes of eukaryotes preserve a large amount of viral sequences derived from both viruses that actively integrate into the host genome and viruses which do not have this capacity but have become integrated incidentally by other means (e.g., nonhomologous recombination or by interaction with retroelements) (2, 3). Over time, the host genome becomes populated by lineages of functional viruses that persist by cycles of reactivation, horizontal transmission, and integration (“intact endogenous viruses”), defective viral sequences that have accumulated inactivating mutations (“viral genomic fossils”), and in some cases, viral sequences that have been repurposed to benefit the host (“viral exaptations”) (3). Study of viral sequences found in host genomes provides insights into the deep origins and evolution of viruses as well as into the ancient interactions between viruses and hosts.

Although sequences derived from every type of viral genome (Baltimore groups) have been discovered integrated in the genomes of animals, retroviruses remain the most thoroughly studied group of endogenous viruses (6). Indeed, endogenous retroviruses account for significant proportions of vertebrate genomes, and their study has led to key evolutionary insights, for example, by shedding light on the evolutionary history of lentiviruses, including the important human pathogen HIV (7, 8). The evolutionary success of retroviruses has been linked to their capacity of integration, latency, and reactivation in host cells. However, they are not the only group of animal viruses with this ability; several families of DNA viruses can also integrate into the host chromosome and are known to transmit vertically. Human herpesviruses 6 and 7 stably integrate into the telomeric regions and have been passed vertically in the human population (9–12). Other less-well-known families of endogenous viruses are the *Teratorns*, which have been found in the genomes of fish (13–15), and the *Mavericks*, which are widely but patchily distributed across eukaryotes and are commonly found in the genomes of aquatic animals (16–19).

*Mavericks* had been widely considered transposons, given that they appear in multiple copies in host genomes and that the details of their molecular biology remain obscure (16, 17). However, both evolutionary analyses and computational modeling of protein structure have shown that they contain a conserved set of genes involved in the formation of virions that encode major and minor capsid proteins, a DNA packaging ATPase, and an adenoviral-like protease (19–21). In addition, *Mavericks* encode a protein-primed family B DNA polymerase (PolB) and a retroviral-like integrase (16, 17). In fact, genome content and phylogenetic analyses firmly nest *Mavericks* within the viral kingdom *Bamfordvirae*, together with other viral lineages such as adenoviruses, virophages, and the nucleocytoplasmic large DNA viruses (20–23). These observations strongly suggest that *Mavericks* are a lineage of endogenous DNA viruses that can occasionally form virions and undergo exogenous transmission (19, 24).

In vertebrates, *Mavericks* have been found in the genomes of teleost fish, coelacanths, amphibians, birds, and nonavian reptiles. They are an ancient lineage of viruses that have infected vertebrates for the last 419 million years (My). Most of the intact endogenous viruses occur in teleost fish (97%), while only 4 (3%) have been found in tetrapod genomes (19). All of the elements found so far in birds seem to be degraded, and they were believed to be altogether absent from the genomes of mammals (16, 17, 19). It was therefore surprising when a *Maverick*-like integration was recently reported on the human chromosome 7 (24, 25). The sequence contained hits to the DNA polymerase, packaging ATPase, and major capsid protein, but other markers such as the integrase and minor capsid were not detected (24). Furthermore, the authenticity of the integration had not been verified, and thus the possibility of spurious sequence contamination had not been ruled out (26). Authenticity can be confirmed by a comparative genomic analysis of the integration locus demonstrating orthology in different species; this shows that the integration was ancestral and suggests that it reached fixation at some point in the past (2, 26).

Here, we conduct a detailed comparative genomic and evolutionary analysis of the chromosome 7 integration locus across mammals and verify the authenticity of the integration via the syntenic arrangement of neighboring protein coding genes in multiple species. We find additional hits to conserved *Maverick* genes, including the minor capsid protein and integrase, which serve to better understand the genome of the ancestor and the origin of the integration. We also discover and authenticate a new element on human chromosome 8 with hits to the DNA polymerase, PM, PZ, and major capsid proteins. Our findings allowed us to reconstruct the evolutionary history of the two human elements and infer their timescales of evolution. We also discovered numerous *polB*-like sequences in marsupials and monotremes, which suggests that *Mavericks* had not gone extinct and were still circulating in the genomes of the stem mammals. Taken together, these results provide new insights into the evolutionary history of *Mavericks* in mammals and the forces that have shaped their evolution. We also discuss several hypotheses that could explain the demise of the elements in birds and mammals.

## RESULTS

We investigated the evolutionary history of the two human *Maverick* insertions initially discovered by local similarity to the DNA polymerase of the endogenous virus found in the genome of the common box turtle, Terrapene carolina. We first identified the closest protein coding genes surrounding the integrations in the human genome, which then allowed us to find the orthologous regions across mammals and to assess whether the integrations were present or absent in these species. With a detailed knowledge of orthology, we used temporal information about the divergence of different host clades to reconstruct a time-calibrated phylogeny of the elements in mammals and to uncover new aspects of their evolutionary history.

We could determine that the *Maverick*-like integrations on chromosomes 7 and 8 are orthologous across the clade of placental mammals (Eutheria). The orthology of the regions was validated by the syntenic arrangement of the closest protein coding genes, the presence of the taxa in the whole-genome alignment of 120 mammals, and the relative arrangement of BLAST hits to different *Maverick* proteins that were consistent with the most common genetic organization observed in vertebrate *Mavericks* (19). In addition, the taxonomic distributions resulting from the BLAST hit and whole-genome alignment methods were consistent with each other (see the Excel file and multiple sequence alignments at https://doi.org/10.6084/m9.figshare.17819708). However, some taxa present in the whole-genome alignment corresponding to the *polB* marker were not detected by the BLAST method, probably because of high divergence of the sequences (producing higher E values than the cutoff). We could not detect orthologous integrations in the genomes of monotremes (Ornithorhynchus anatinus) or marsupials either through BLAST or in the whole-genome alignments.

**FIG 1:**
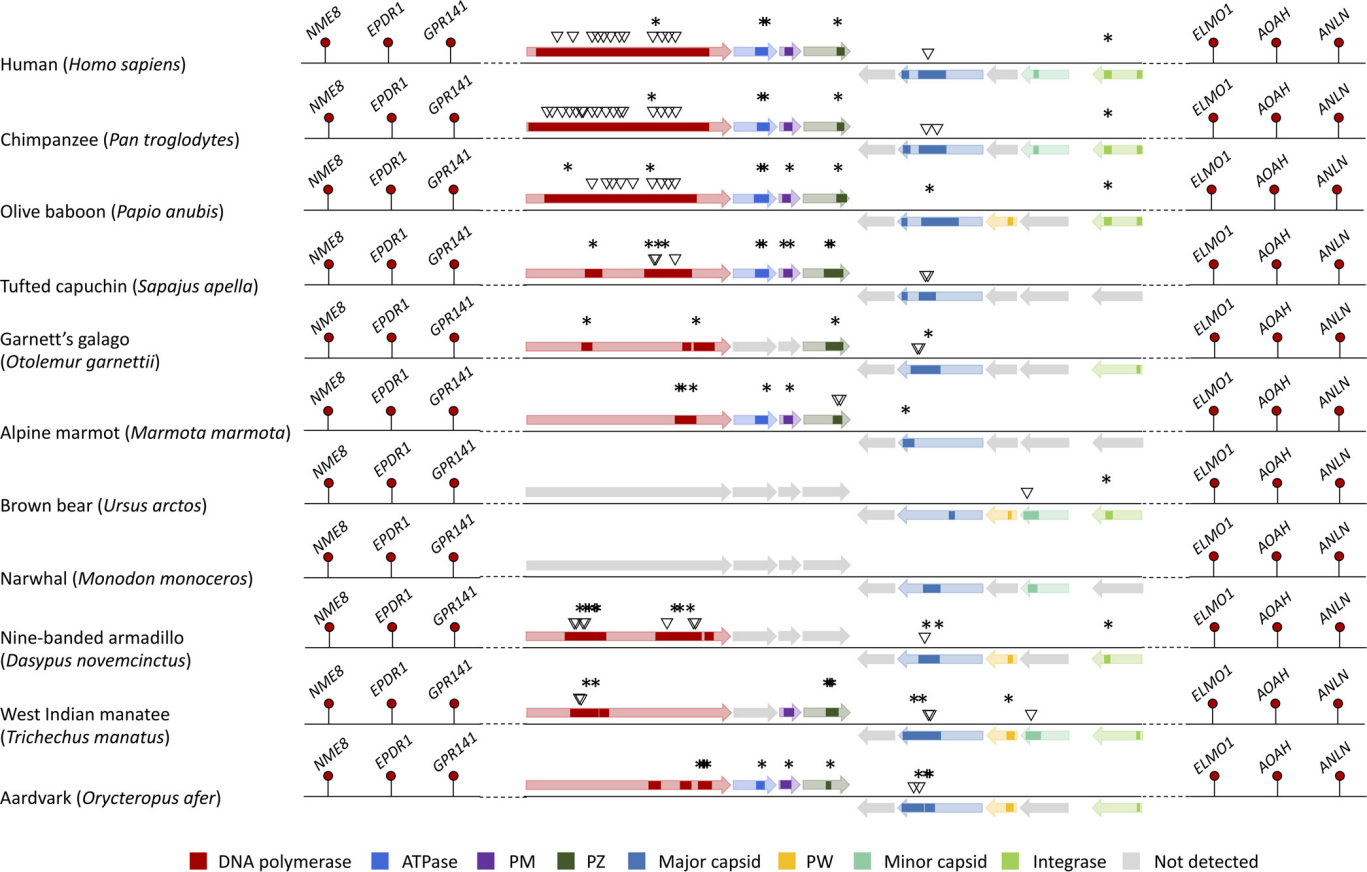
Comparison of the regions orthologous to the chromosome 7 *Maverick* integration in humans (Homo sapiens). Colored rectangles denote regions of local similarity to the proteins encoded by the common box turtle *Terrapene carolina* (tBLASTn, E value of <0.05). Asterisks represent predicted early stop codons, and inverted triangles indicate frameshift mutations (inferred from the BLAST local alignments and modeling of the gene structure in GeneWise [59]). The thick horizontal arrows represent the open reading frames found in the intact *Maverick* of the turtle. Hits to eight of the nine conserved proteins found in vertebrate *Mavericks* could be detected across the orthologues (missing hits to the protease).

**FIG 2:**
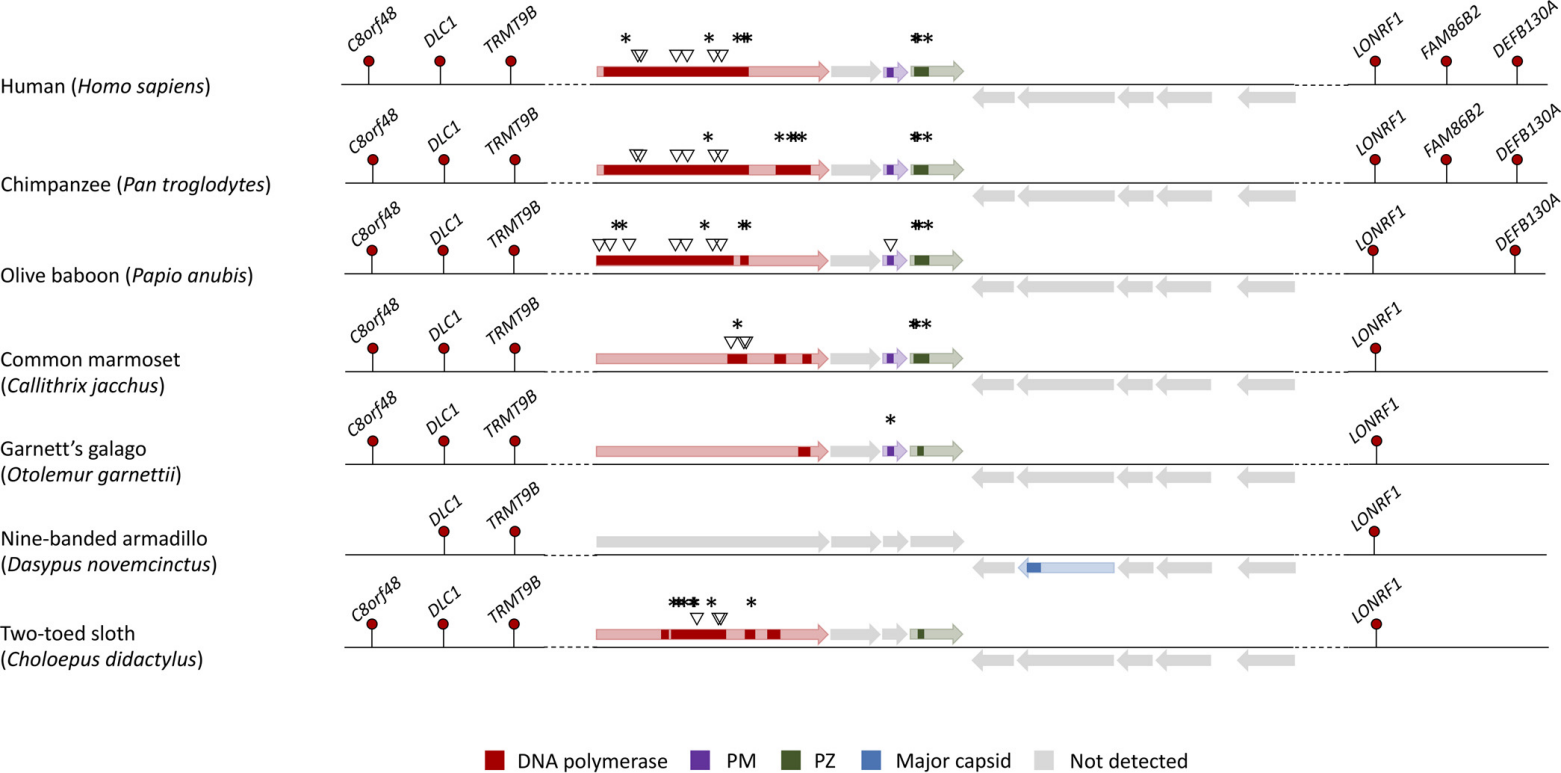
Comparison of the regions orthologous to the chromosome 8 *Maverick* integration in humans (Homo sapiens). Colored rectangles denote regions of local similarity to the proteins encoded by the common box turtle *Terrapene carolina* (tBLASTn, E value of <0.05). Asterisks represent predicted early stop codons, and inverted triangles indicate frameshift mutations (inferred from the BLAST local alignments and modeling of the gene structure in GeneWise [59]). The thick horizontal arrows represent the open reading frames found in the intact *Maverick* of the turtle. Only four of the nine conserved proteins in vertebrate *Mavericks* could be detected, indicating more extensive degeneration than that of the chromosome 7 group of orthologues.

Orthologues of the human chromosome 7 element were found in the genomes of primates, rodents, afrotherians (elephants, manatees, and aardvark) and xenarthrans (sloths and armadillos) (Fig. 1). In addition, the *polB* marker was present in the whole-genome alignment for scandentians (tree shrews), dermopterans (flying lemurs), lagomorphs (hares), and two laurasiatherians: the star-nosed mole (Condylura cristata) and the European hedgehog (Erinaceus europaeus). This finding is consistent with the presence of hits to the major capsid, minor capsid, PW, and integrase in other laurasiatherians, namely, carnivores and cetaceans (Fig. 1; see also the Excel file at https://doi.org/10.6084/m9.figshare.17819708). As expected for a *Maverick*, the hits to the integrase are on the same strand as the major capsid and did not show similarity to the integrases of retroelements in Censor (27). In total, we found hits to 8 of the 9 conserved core proteins reported for vertebrate *Mavericks* in the orthologues of the human chromosome 7 integration (Fig. 1). Orthologues of the human chromosome 8 integration were found in the genomes of primates and xenarthrans. The *polB* marker again had a broader taxonomic distribution in the whole-genome alignment and also included scandentians, dermopterans, lagomorphs, and bats (Fig. 2). The chromosome 8 element is more degraded than the chromosome 7 *Maverick*, and we could detect only 4 hits to the set of 9 conserved core proteins (Fig. 2). Most elements preserve fragments of the DNA polymerase, PM, and PZ genes, while a single element in the nine-banded armadillo (Dasypus novemcinctus) showed a hit to the major capsid protein (Fig. 2).

All the integrations were highly degraded, showing strong signs of being nonfunctional at the protein level. The open reading frames are interrupted with multiple early stop codon and frameshift mutations (Fig. 1 and 2). The elements also lack the terminal inverted repeats characteristic of intact *Maverick* elements. In addition, the genetic distances of the elements are not significantly different from the distances between noncoding regions in their surrounding genomic neighborhood (Kolmogorov-Smirnov test, *P* > 0.99) (see Fig. A1 and A2 at https://doi.org/10.6084/m9.figshare.17819708), which suggests that the elements have been evolving neutrally. Interestingly, the chromosome 7/8 *Maverick* orthologues appear to have been lost in some mammals, since they could not be detected by the BLAST or whole-genome alignment method but were still present in related species. For example, the chromosome 7 integration appears to be absent from the genome of the Ugandan red colobus (Piliocolobus tephrosceles), while it is present in other colobine monkeys (Trachypithecus francoisi and Rhinopithecus roxellana). Similarly, the chromosome 8 integration seems to be absent from the genomes of the gray mouse lemur (Microcebus murinus) and Coquerel’s sifaka (Propithecus coquereli), despite the presence of the *polB* marker in the northern greater galago (Otolemur garnettii) (this sequence is present in the whole-genome alignment and shows a BLAST hit to the PolB of the common box turtle with an E value of 5e−4).

**FIG 3:**
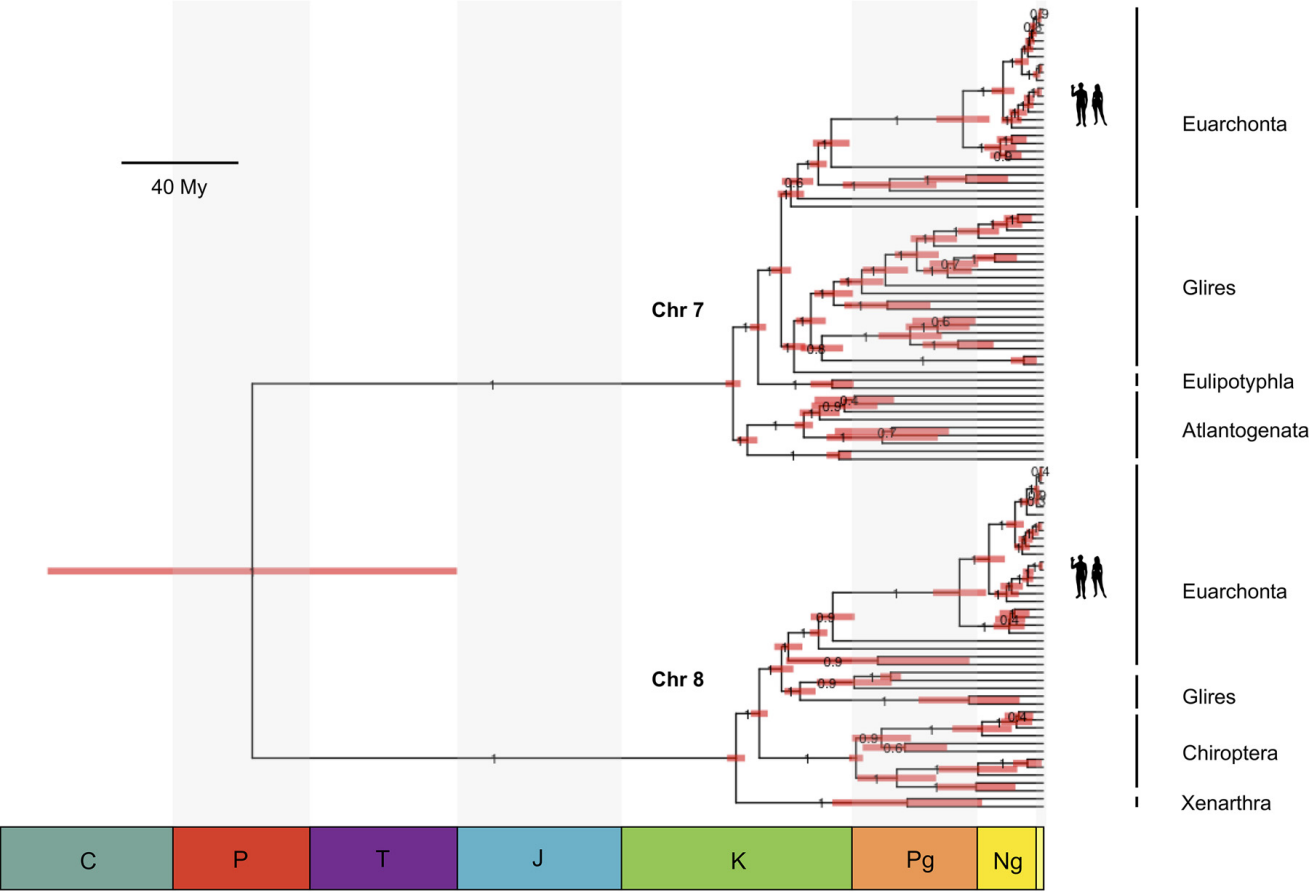
Phylogenetic tree of the mammalian polymerase B-like sequences orthologous to the viral fossils in human chromosomes 7 and 8 (nucleotide data). The horizontal node bars represent the 95% highest probability densities (HPD) for the age of the ancestor. Nodes show posterior probabilities of the clades. The scale bar is given in million years (My). C, Carboniferous; P, Permian; T, Triassic; J, Jurassic; K, Cretaceous; Pg, Paleogene; Ng, Neogene.

The presence of the *polB* marker on both the chromosome 7 and 8 orthologues allowed us to estimate a joint phylogeny with the common ancestor of the elements represented by the root of the tree (Table 1 and Fig. 3; see also Fig. A3 at https://doi.org/10.6084/m9.figshare.17819708). By using the nucleotide data, the minimum dates of the human insertions were estimated at 103 (100 to 105) million years ago (Mya) for the chromosome 7 element and 102 (99 to 105) Mya for the chromosome 8 element. Almost the same estimates were obtained using the protein data, i.e., 102 (100 to 105) Mya for the chromosome 7 integration and 102 (99 to 105) Mya for the chromosome 8 integration. These ages correspond to the transition between the Early and Late Cretaceous. The inferred age of the most recent common ancestor (MRCA) of the two integrations was more variable between the nucleotide and protein data but was also of the same order of magnitude. By using the nucleotide data, the root of the tree was estimated to be at 262 (200 to 335) Mya, suggesting that the ancestor could have existed from the end of the Paleozoic Era to the start of the Mesozoic Era. The protein data suggest a more recent age of the ancestor, placing it at 127 (101 to 167) Mya, between the Jurassic and Cretaceous Periods.

**TABLE 1:** Posterior estimates for the age of the root and minimum ages of the chromosome 7/8 integrations

Node	Data	Inferred age (Mya from the present)		95% HPD^*a*^ (Mya from the present)
		Mean	Median	
Root^*b*^	Nucleotide	262	257	200–335
	Amino acid	127	121	101–167
Chromosome 7 MRCA^*c*^	Nucleotide	103	103	100–105
	Amino acid	102	102	100–105
Chromosome 8 MRCA^*d*^	Nucleotide	102	102	99–105
	Amino acid	102	102	99–105

a 95% highest probability density of the posterior distribution.

b MRCA of the chromosome 7/8 paralogues.

c MRCA of the chromosome 7 group of orthologues.

d MRCA of the chromosome 8 group of orthologues.

The rate of nucleotide evolution that we inferred in our analysis (mean = 0.0026; interquartile range [IQR] = 0.0021, 0.0029) agrees with the overall pangenomic substitution rate reported previously for mammals of 0.0027 nucleotide substitutions/site/My (28), which is an independent confirmation that the timescales were calibrated properly. The evolutionary rate of amino acids of 0.007 amino acid substitutions/site/My (IQR = 0.0066, 0.0076) is about twice the rate reported previously for other vertebrate *Mavericks* (19), so the age of the ancestor estimated by the amino acid data may represent a more conservative estimate.

In addition to characterization of these two placental mammal-wide orthologues, we discovered multiple hits to the box turtle *Maverick* polymerase in nonorthologous regions in the genomes of marsupials and egg-laying mammals (monotremes). We found 40 hits to the *Maverick* DNA polymerase in the genomes of 7 marsupials, from both the American and Australian continents (see Table A2 at https://doi.org/10.6084/m9.figshare.17819708). Three hits were found in the genomes of egg-laying mammals, one in the platypus (*Ornithorhynchus anatinus*) and two in the short-beaked echidna (Tachyglossus aculeatus) (see Table A2 at https://doi.org/10.6084/m9.figshare.17819708).

## DISCUSSION

Our analyses have revealed two ancient integrations from *Mavericks* in the human genome that are shared across the placental mammals. Critically, we have established orthology of the two integrations across placental mammals by describing the conserved arrangement of protein coding genes around the integration loci (conserved synteny). The discovery of orthologous integrations to the human chromosome 7 and 8 elements in other mammals is strong evidence for their authenticity and rules out possible sequence contamination and misassembly (26). Therefore, we were able to authenticate the two human *Maverick* elements and confirm the existence of *Maverick* integrations in the genomes of placental mammals. This changes our view of the evolutionary history of *Mavericks* in vertebrates, which were thought to be entirely absent from the mammalian lineage (16–19).

There is a blurry boundary between viruses and transposons (3, 5), and one possibility is that the two integrations we describe originated from a transposon that evolved from *Mavericks*. Before it was realized that they contain all the genes necessary for capsid formation (20, 21), and that these genes are under strong purifying selection (19), *Mavericks* were considered transposons (16, 17). In the human chromosome 7 group of orthologues, we found remnants of the DNA packaging ATPase, major and minor capsid proteins, in addition to DNA polymerase and integrase, and another three core proteins also present in the *Mavericks* of vertebrates. These findings suggest that the ancestor of the element contained the genes necessary to form a virion and integrated as an endogenous virus. Similarly, we found a fragment of the major capsid in the chromosome 8 orthologue in the genome of the nine-banded armadillo. If these two integrations had been derived from a *Maverick*-like transposon, we would expect natural selection to have deleted the genes responsible for capsid morphogenesis, since these would serve no purpose. We also would have expected to find a greater copy number of the elements and perhaps an intact element with inverted repeats required for mobilization. However, our findings are more consistent with an integrated viral genome that has progressively degenerated in their hosts. Therefore, these two integrations are clearly highly degraded endogenous viral elements (EVEs), not transposon sequences.

We estimated minimum ages for the chromosome 7 and 8 integrations of 103/102 (100 to 105) My and 102 (99 to 105) My, respectively, which represent to our knowledge the oldest nonretroviral EVEs found in the human genome. An endogenous retrovirus L element (ERV-L) of a comparable age (at least 104 to 110 My) has also been identified in humans and shown to be orthologous across the placental mammals (29). Comparison of the sequence divergence between the 5′ and 3′ long terminal repeats (LTRs) of elephant and human (the 5′ and 3′ LTRs would be identical upon integration in the common ancestor) further suggests that the element may have integrated 24 to 36 My prior to the initial split between afrotherians and boreoeutherians (29). In terms of DNA viruses, an endogenous parvovirus-like element discovered in an intron of the *Ellis-van Creveld syndrome 2* gene was shown to be present in primates, carnivores, ungulates, and dolphins but not in afrotherians, giving it a minimum age of 98 My (30). The human genome thus preserves remnants of multiple viruses that infected our ancestors during the Mesozoic Era.

Discovery of the human element found on chromosome 8, together with the one found on chromosome 7, allowed us to gain a more detailed understanding of the evolutionary history of the elements before the diversification of placental mammals. Our time-calibrated phylogeny with both elements allowed us to infer the age of the root. This analysis suggests that *Mavericks* were circulating in the ancestors of mammals from the end of the Paleozoic to the Jurassic/Cretaceous Periods. Further evidence for this is the discovery of multiple *polB*-like sequences at nonorthologous positions in the genomes of marsupials and egg-laying mammals, which indicates that active *Mavericks* infected the MRCA of mammals. Clear orthology of the chromosome 7 and 8 integrations across placental mammals together with their absence in marsupials and monotremes suggests that the viruses continued to be active and integrated into the genome of the placental ancestor after the split with marsupials. Molecular estimates place this divergence around 172 (168 to 178) Mya (31), which is consistent with the age of the earliest fossil eutherian, Juramaia sinensis, from the Late Jurassic (160 Mya) of China (32, 33). Thus, it seems highly likely that active *Mavericks* persisted in the genomes of the early eutherian ancestors to the end of the Jurassic Period.

The extensive degeneration of the chromosome 7 and 8 integrations also suggests that they had already been inactivated by the time of the MRCA of placentals (~102 Mya) (34), pointing to an older age of integration. This is evidenced by deletion of the genes downstream of the PZ gene in most chromosome 8 orthologues and the absence of the protease gene (*pro*) in the chromosome 7 insertions. The two *Maverick* insertions thus resemble those found in birds, which are highly degraded and whose genes contain multiple inactivating mutations (19). We show that the two insertions found in humans and placental mammals are likely to be nonfunctional, since they do not seem to be under selection, they do not localize to PIWI-interacting RNA (piRNA) clusters, and they have been lost on several occasions in several species of mammals.

The reasons for the demise of *Mavericks* in mammals and birds remain a mystery. Here, we hypothesize several plausible scenarios. It has been suggested that *Maverick*s may function as a virophage-induced defense against the infection of large DNA viruses (35, 36), in particular iridoviruses (19). Iridoviruses are important pathogens of fish, amphibians, and nonavian reptiles, but they do not seem to infect either birds or mammals (37, 38). One possibility is that iridoviruses went extinct in mammals and birds as a result of this *Maverick* defense system. Once their viral hosts went extinct, endogenous virophages (which depend on a host virus for replication) would not have been able to mobilize and this would have led to their degeneration. This hypothesis could be tested by performing iridovirus infection experiments on cell cultures of hosts which carry intact *Maverick* elements (teleost fish, amphibians, or nonavian reptiles). Alternatively, endogenous *Mavericks* could have been coopted as a defense against exogenous counterparts. Exaptation of endogenous viruses to serve this purpose has been reported extensively, for example, in the cases of the *ev3*, *ev6*, and *ev9* genes in chicken (39), the *Fv1* and *Fv4* genes in mice (40–43), and the Jaagsiekte endogenous retrovirus (enJS56A1) in sheep, which are able to restrict exogenous viruses (44). Evolution of an effective antiviral defense system would lead to the extinction of the exogenous virus and degeneration of the defense locus once the selective pressure from the pathogen has been lifted (45). Therefore, the *Maverick* integrations found in humans and other placentals could represent an antiviral defense system that has served its purpose and is now decaying. However, *Mavericks* have not been linked to any specific pathology, and their exogenous stage has yet to be observed. Still another possibility is that these *Mavericks* were incidentally inactivated by host innate immune genes that evolved to fight other viral pathogens.

In addition to the viral arms race scenarios, other hypotheses relating to changes in host biology could also explain the demise of *Mavericks* in the genomes of mammals and birds. It is possible that the receptor used for entry has been lost or has acquired resistance mutations in these animals and the elements can no longer amplify in the germ line by reinfection or cross-species transmissions. Support for this idea would require identification of the host cell receptor used for the entry of *Mavericks* and assessment of its present state across vertebrates, both of which are still unknown. Finally, the demise of *Mavericks* in these lineages might be linked to terrestrialization and the origin of the amniotic egg, which may have limited the opportunities for the spread of these viruses and led to their ultimate degeneration. Although the natural history of *Mavericks* is still not understood, it seems *Mavericks* are most successful in aquatic organisms, which suggests a waterborne mode of transmission (18, 19), and perhaps they become active at the early stages, when sperm and ova are released into the water by animals with external fertilization. In fact, potentially active elements reach their highest diversity and copy numbers in fish (where cross-species transmissions have also been detected), while only two intact elements have been discovered in amniotes (in turtles and lizards) (19, 24). It therefore seems likely that the reduced opportunities for transmission could have led to a decrease in copy numbers, which eventually made *Mavericks* prone to extinction.

Our results support a model where *Maverick* endogenous viruses circulated in the genomes of the stem mammals at the end of the Paleozoic Era and remained active in eutherian ancestors into the Jurassic/Cretaceous Periods. Therefore, the elements found integrated on human chromosomes 7 and 8 represent the relics of ancient viruses that infected our ancestors >102 Mya. Several hypotheses may explain the demise of *Mavericks* in mammals, including exaptation for an antiviral defense system followed by decay, inactivation by an innate immune mechanism, and loss/mutation of the host cell receptor or changes to the biology of the hosts that lowered their chances of transmission. However, based on the study of these two loci, it is not possible to definitely rule out the existence of active *Mavericks* in the genomes of all mammals. Further sequencing of mammalian genomes and characterization of their *Mavericks* can provide answers to this question. Experimental studies focusing on the molecular virology of *Mavericks* and the virus-host interactions are also needed to shed light on these various issues.

## MATERIALS AND METHODS

We used the protein-primed polymerase B (PolB) of the common box turtle (*Terrapene carolina*) *Maverick* (19, 24) as a probe to screen the human genome with tBLASTn (46) (assembly GRCh38.p13; BLOSUM 45 substitution matrix; word size, 2). We obtained two hits with E values of <1e−7: one on chromosome 7 (chr7:37601124–37601615; query cover, 15%; E value, 8e−8; percent identity, 31.14%) and another on chromosome 8 (chr8:12858023–12858613; query cover, 25%; E value, 5e−8; percent identity, 29.95%). In order to assess the orthology of the integrations in the genomes of other mammals, we initially tried to use nucleotide flanks and find pairs of hits in other genomes, but this approach was not successful due to extensive sequence degeneration. To overcome this limitation, we identified the three most proximal protein coding genes upstream and downstream of the integrations by using the Ensembl genome browser (47). The sequence conservation of the genes allowed us to analyze synteny and orthology across mammals.

The protein coding genes used as genetic landmarks for the chromosome 7 integration were *NME8*, *EPDR1*, *GPR141*, *ELMO1*, *AOAH*, and *ANLN*, and those for chromosome 8 were *C8orf48*, *DLC1*, *TRMT9B*, *LONRF1*, *FAM86B2*, and *DEFB130A* (NCBI protein database [48]). We used these sequences as queries in tBLASTn (set to the default settings) to screen the NCBI RefSeq genome database using the following taxon labels: “Monotremata (taxonomy identification number [taxid] 9255),” “Metatheria (taxid 9263),” “Afrotheria (taxid 311790),” “Xenarthra (taxid 9348),” “Laurasiatheria (taxid 314145),” and “Euarchontoglires (taxid 314146).” To verify the presence or absence of the integration, we screened the regions flanked by the genetic landmarks with the full set of proteins encoded by the four tetrapod *Mavericks* reported previously (19) again with tBLASTn (E value, <1e−5).

As an independent test for the presence of the integrations, we used the whole-genome alignment of 120 mammals published by Hecker and Hiller (49). We extracted the regions corresponding to the human *polB* integrations and extended them by assuming that the ancestral gene coded for 1,053 amino acids as in the box turtle *Maverick* (chr7:37600749–37603904, chr8:12856919–12860047). The regions were extracted from the chromosome 7 and chromosome 8 whole-genome alignments in MAF format (85.16 GB and 81.07 GB, respectively) with custom *.bed files and using the mafsInRegion utility (50). Subalignments were transformed into fasta format with the MAF to FASTA program (version 1.0.1) in Galaxy (51). The taxa present in these subalignments were then compared to those obtained by the BLAST hit method.

We merged the chromosome 7 and 8 subalignments corresponding to the *polB* gene by using the –merge function in MAFFT (52). The alignment was trimmed in trimAl version 1.4.rev22 (53) with the –automated1 option (which selects the optimal trimming method for the alignment). We then selected the best nucleotide substitution model in ModelTest-NG version 0.1.7 (54) (TVM + Γ4 under the Akaike information criterion [AIC], Bayesian information criterion [BIC] and size-corrected AIC [AICc]. After determining the orthology of the integrations, we conducted a phylogenetic analysis under a relaxed molecular clock using divergence-time calibrations in BEAST 2 version 2.6.7 (55). For this, we used 13 calibration points obtained from TimeTree (34) (see Table A1 at https://doi.org/10.6084/m9.figshare.17819708) using normal distributions and then ran a Bayesian Markov Chain Monte Carlo (MCMC) for 100,000,000 generations, sampling every 5,000th generation. We confirmed that the analysis had converged by inspecting the mixing and stationarity of posterior samples and ensuring that the estimated sample sizes (ESSs) were greater than 200 for all parameters (burn-in of 25%). An additional time-calibrated phylogeny was inferred based on a subset of predicted amino acid sequences for the PolB and run for 50,000,000 generations until convergence was attained (model JTT+G4).

Finally, we tested the possibility of selective constraints acting on the integrations. To this end, we calculated the pairwise genetic distances (measured as the observed proportion of nucleotide differences) between the taxa in the chromosome 7 and chromosome 8 subalignments separately and compared these to 100 randomly sampled surrounding noncoding genomic regions of the same size in each case. We built two empirical distributions from these and then tested the hypothesis that the distribution of *Maverick* genetic distances was distinct from the noncoding distribution. This was done with a one-tailed, nonparametric Kolmogorov-Smirnov test. An R script was developed for this purpose using functionality from the Ape package (56, 57). We further checked to see if the human integrations fell within piRNA clusters reported for chromosomes 7 and 8 (58), but this resulted in no matches (see the Excel file at https://doi.org/10.6084/m9.figshare.17819708).

### Data availability.

Additional data for this study, i.e., Fig. A1, A2, and A3, Tables A1 and A2, and an Excel file (Tables S1, S2, and S3), have been deposited in Figshare (https://doi.org/10.6084/m9.figshare.17819708); the nucleotide and amino acid multiple sequence alignments are also available in Figshare. The R Code written to compare distributions of pairwise genetic distances is available on GitHub (https://github.com/josegabrielnb/pair-wise_distributions).
